# Molecular Dynamics Simulation of Plasticizing Effect of Mixed Dioctyl Phthalate and Isosorbide Diheptanoate on Polyvinyl Chloride Material

**DOI:** 10.3390/polym17121655

**Published:** 2025-06-14

**Authors:** Qin Lei, Xijian Yi, Wenxi Yu, Juan Cheng, Siyu Ou, Qiong Xue, Haiyun Jiang

**Affiliations:** 1 School of Packaging Engineering, Hunan University of Technology, Zhuzhou 412007, China; m230805z1003@stu.hut.edu.cn (Q.L.); m22085600024@stu.hut.edu.cn (X.Y.); m24080500009@stu.hut.edu.cn (S.O.); xueqiong@hut.edu.cn (Q.X.); jianghaiyun@hut.edu.cn (H.J.); 2 College of Packaging Engineering, Jinan University, Zhuhai 519070, China; chengj2008@jnu.edu.cn

**Keywords:** molecular dynamics simulation, PVC, isosorbide diheptanoate, dioctyl phthalate, glass transition temperature

## Abstract

A molecular dynamics simulation was adopted to investigate the plasticizing effect of polyvinyl chloride (PVC) and its mechanism by blending isosorbide heptylate (SDH) with the traditional plasticizer dioctyl phthalate (DOP) and to explore the feasibility of SDH partially replacing DOP in PVC film. The results demonstrated that the difference in the solubility parameter between SDH and PVC was smaller than that between DOP and PVC, indicating the superior compatibility of SDH with PVC. This enhanced compatibility was further supported by the significantly higher interaction energy between SDH and PVC compared to that between DOP and PVC, primarily attributed to the stronger interactions formed between the polar functional groups in the SDH molecules and the PVC’s molecular chains. The analysis of the glass transition temperature demonstrated that the plasticizing effect of the SDH/DOP mixed plasticizer on the PVC exhibited intermediate behavior between that of pure SDH and DOP systems, showing a decreasing trend with an increasing proportion of SDH. An analysis of the radial distribution function further confirmed that the probability of hydrogen bond formation between the SDH and PVC molecules was significantly higher than that between DOP and PVC, contributing to the strong interaction between the SDH and PVC. From the analysis of the plasticizer’s diffusion, it was clearly concluded that the migration resistance of SDH was superior to that of DOP. These research findings can provide fundamental data and guidance for the strategy of partially replacing DOP with SDH.

## 1. Introduction

Polyvinyl chloride (PVC), one of the world’s three major synthetic plastics, is widely used in electrical insulation, building materials, food packaging, cosmetics packaging, medical equipment, and many other areas [1,2]. However, due to the presence of highly polar chlorine atoms and strong intermolecular forces in PVC, it exhibits high strength but low toughness, along with a high glass transition temperature (*T*_g_), which restricts its development and widespread application [3]. To address these limitations, PVC resin is typically blended with plasticizers to improve its flexibility and processability [4]. Traditionally, phthalates (PAEs), such as dioctyl phthalate (DOP), have constituted the predominant class of commercial plasticizers, attributable to their exceptional plasticization efficacy and cost-effectiveness. With the implementation of the global “plastic restriction order” legislation, the application domains of PAEs have faced increasing restrictions [5]. The migration of plasticizer molecules not only undermines the long-term mechanical stability of PVC but also raises significant health concerns due to their intrinsic toxicity [6]. Consequently, developing non-toxic plasticizers with excellent thermal stability, a superior performance, and high migration resistance for PVC remains a substantial challenge.

Bio-based plasticizers have attracted considerable attention and high expectations from both the scientific community and industry. Jia et al. [7] successfully reduced the *T_g_* of PVC by grafting cashew phenol onto the PVC chain, thereby enhancing the toughness of the synthetic material while ensuring no migration of the plasticizer was observed. Subsequently, Jia et al. [8] extended this approach by grafting cardanol glycidyl ether, epoxidized acetylated castor oil methyl ester, and epoxidized soybean oil onto PVC, resulting in flexible PVC materials. This study demonstrated a significant reduction in *T*_g_ from 82.9 °C to 42.1 °C, while the bio-based synthetic plasticizers exhibited excellent migration resistance, with no detectable migration after prolonged immersion in n-hexane for 2 h.

Other effective graft modifiers include trichloro-s-triazine [9], salicylic acid and epoxidized fatty acid methyl esters [10], ricinoleic-acid-derived phosphate ester [11], and 4-fluoro thiophene [12], all demonstrating enhanced PVC flexibility and migration resistance.

It is crucial to emphasize that although the aforementioned chemical methods can enhance the plasticity of PVC, the grafting process necessitates specific chemical reactions tailored to each raw material, thereby limiting their industrial applicability. Given these process limitations, researchers are prioritizing the direct incorporation of synthetic bio-based plasticizers into PVC substrates via physical blending to evaluate their plasticizing effects. Zhao et al. [13] demonstrated that the addition of 1,2-cyclohexane dicarboxylic acid di (trimethylene glycol methyl ether) ester to PVC films effectively suppressed plasticizer migration while enhancing the elongation at break and the initial thermal decomposition temperature. Bio-based hyperbranched polyethers, synthesized in a one-pot reaction from triethyl citrate, glycerol triacetate, and acetic anhydride, exhibited a lower plasticizing effect compared to that of DOP but provided superior thermal stability, reduced solvent extractability, and enhanced resistance to volatility. Through innovative synthesis of isophorone diisocyanate and butyl lactate, Qian et al. [14] developed a novel plasticizer demonstrating an excellent plasticizing performance and migration resistance, highlighting its commercial viability. Furthermore, recent studies have identified multiple effective alternatives, including acetylated citric acid [15], dioctyl phthalate di(ethylene glycol ether) [16], di-butyl tartrate with varying alkyl chain lengths [17], and citrate esters [3], with all exhibiting an excellent plasticization efficiency relative to that of traditional phthalates.

The aforementioned bio-based plasticizers exhibit significant plasticizing effects; however, their widespread industrial application remains constrained by high production costs and limited yields. Reducing the formulation materials or adopting partial substitution methods is critical for realizing their commercial potential. Luo et al. [18] reported that bio-based plasticizers consisting of two epoxidized cardanol esters with varying epoxy contents and alkyl chain lengths exhibited synergistic plasticizing and thermal stabilization effects on PVC materials containing DOP.

Li et al. [19] revealed that incorporating isosorbide diheptanoate (SDH) into PVC films significantly enhances their flexibility, suppresses plasticizer migration, and improves both the elongation at break and initial thermal decomposition temperature. Notably, SDH demonstrated a superior thermal stability, a reduced volatility, and a lower solvent extractability compared to those of DOP, indicating its viability as a sustainable substitute. Nevertheless, the fundamental mechanisms underlying partial substitution remain elusive, impeding the development of optimized formulations. Recent advancements in computational science have facilitated the systematic implementation of molecular dynamics (MD) simulations to probe the molecular-level interaction mechanisms in materials [3,20,21], thereby enabling predictive design of optimized experimental protocols. Li et al. [22] conducted MD simulations to investigate the thermodynamic capacitance, plasticization efficiency, and molecular mobility in PVC/plasticizer systems incorporating phthalates, terephthalates, trimellitates, and citrates. Several studies have systematically quantified the plasticizing efficacy of bio-derived plasticizers, such as octyl decyl succinate [23], acetylated citric acid [15], and alkyl-chain-tailored citrate esters [3].

Building upon these computational advancements, this study applies MD simulations to comprehensively assessing the compatibility, *T*_g_, mechanical properties, interaction energy (*E*_Inter_), fractional free volume (FFV), and radial distribution function (RDF) of PVC blends containing both SDH and DOP, with the objective of providing a theoretical basis for partially replacing DOP with SDH in practical applications.

## 2. Simulation Methods

### 2.1. Cell Construction

**Figure 1:**
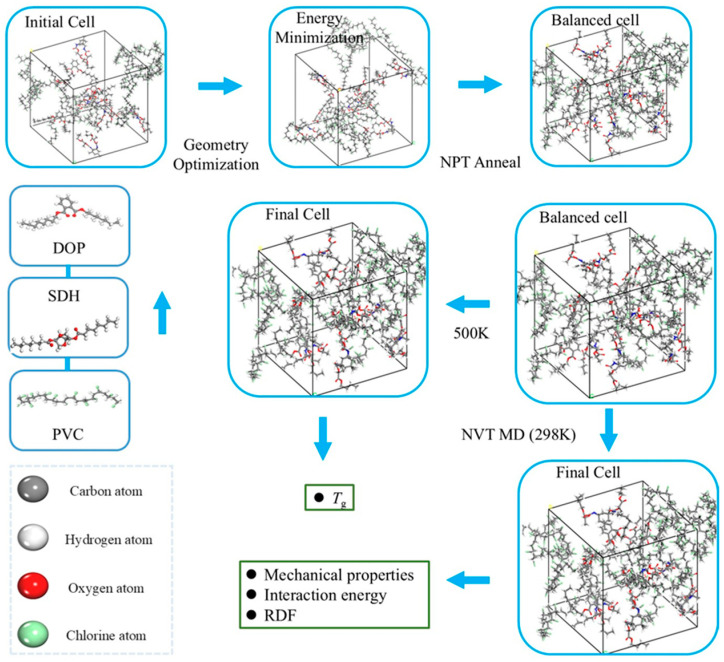
A schematic diagram of cell building and simulation.

A PVC polymer chain comprising 100 repeat units [24], along with discrete molecules of DOP and SDH, was modeled using the “Amorphous Cell” module in Materials Studio(version 7.0, Accelrys Inc., San Diego, CA, USA). Structural relaxation of the PVC chain and plasticizers was performed via the “Smart” method for 100,000 minimization steps to achieve energy minimization. Independent simulation cells were built with periodic boundary conditions to eliminate surface effects using the “Amorphous Cell” module. The initial density of each cell was set to 0.5 g/cm^3^. The plasticizer content constitutes 30% of the total mass of the polymer. Figure 1 schematically depicts the modeling workflow, while Table 1 quantitatively details the simulation systems, including their chemical compositions and key parameters. And chemical structures of DOP, PVC, and SDH are presented in Figure S1.

### 2.2. The Simulation Analysis

Following the construction of the amorphous cells, geometric optimization was performed at a medium level of quality, allowing a maximum of 100,000 steps to ensure convergence. This process significantly minimized the total potential energy of the systems, thereby improving their structural integrity for subsequent simulations. Subsequently, a thermal annealing protocol was then implemented in the NPT ensemble (at a constant pressure and temperature) under the following conditions: each system was heated to 600 K, followed by stepwise cooling to 300 K in 50 K decrements over 200 ps intervals. During each cycle, an NPT ensemble simulation was conducted at a 1 bar pressure and using a 1 fs time step. The heating–cooling cycle was repeated 10 times to ensure structural relaxation (Figure S2). Furthermore, the density of the PVC-DOP/SDH unit cell decreased from 1.41 g/cm^3^ to approximately 1.20 g/cm^3^, whereas the average density of the plasticized unit cell showed minimal variation (Table 1).

Previous studies by Pan et al. [25] on the self-diffusion coefficients of plasticizers employed 10 ns NPT ensemble simulations, demonstrating that extended simulation durations significantly enhance the system equilibration and improve the reliability of the computational results. A study by Olowookere et al. [24] demonstrated that the characteristic properties of most polymers converge at chain lengths of approximately 100–120 repeating units, and they subsequently performed an RDF analysis using simulation data collected within a 12-nanosecond timeframe. Based on this, in subsequent research [26], Olowookere also studied the interaction between polyvinyl chloride (PVC) and its additives in traditional solvents and bio-based solvents. Building upon this empirical evidence, the annealed cells (Figure S3) underwent a 20 ns MD simulation in an NVT ensemble (a canonical ensemble) at 298 K, with a time step of 1 fs. Electrostatic interactions were calculated using the particle–particle particle–mesh (PPPM) method with a 12.5 Å cut-off, while van der Waals interactions were treated via an atom-based approach. The COMPASS II force field was assigned to all molecules. The output files were analyzed to evaluate the RDF, *E*_Inter_, mechanical properties, mean squared displacement (*MSD*), and plasticizer migration resistance. During the stage of the MD simulation (0–20 ns), the temperature and energy fluctuations in the cells at 298 K were maintained within a stable range of 2%. This indicated that the system had achieved equilibrium during this period, as depicted in Figure S4. To determine the *T*_g_ values of the cells, the annealed cells were first subjected to a 2 ns NVT MD simulation at 500 K, followed by a gradual cooling process to calculate *T*_g_.

## 3. Results and Discussion

### 3.1. The Glass Transition Temperature

*T*_g_ is a critical parameter governing polymer performance, as it dictates both the processing temperature and the operational temperature range, thereby serving as a key metric for evaluating commercial viability. Below *T*_g_, strong intermolecular interactions between PVC chains restrict the molecular mobility, resulting in a low kinetic energy and a glassy state characterized by slow specific volume expansion with an increasing temperature [27]. Above *T*_g_, the polymer transitions to a rubbery state, accompanied by weakened chain interactions, increased molecular kinetic energy, and accelerated specific volume growth. This transition reflects the structural shift from restricted segmental motion in the glassy state to enhanced molecular mobility in the rubbery phase [28,29].

**Figure 2:**
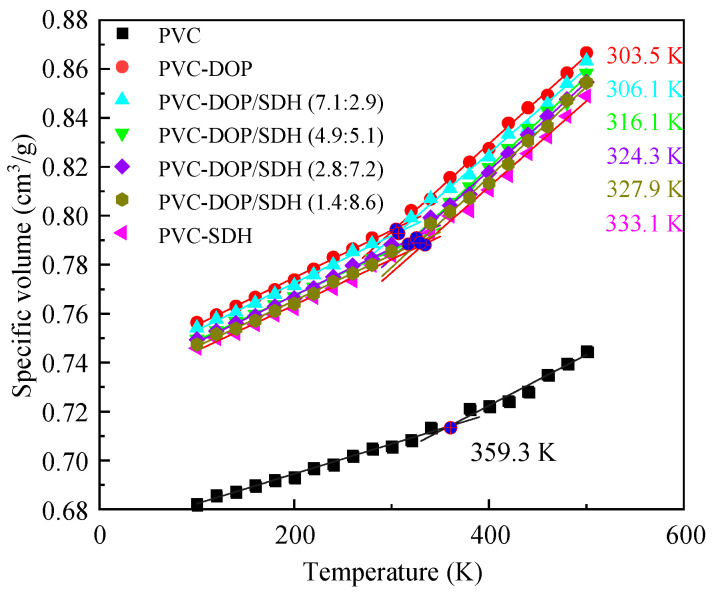
The curves of the specific volume versus temperature for the cells.

Figure 2 presents the specific volume–temperature curves for the cells, with the calculated data fitted using linear regression. The curve for pure PVC exhibits distinct glass transition behavior, yielding a simulated *T*_g_ of 359.3 K. As shown in Table 2, this value aligns with the reported computational results and falls within the experimental ranges for *T*_g_. The discrepancies between our simulated *T*_g_ values and those from the other literature, as well as the experimental data, can likely be attributed to differences in the simulation methodologies, force field parameters, and system configurations. Variations in the polymer chain length, equilibration protocols, or computational conditions (e.g., pressure, temperature ramp rates) may lead to shifts in the predicted *T*_g_. Additionally, the inherent approximations in molecular dynamic force fields, particularly in modeling the van der Waals interactions and chain dynamics, could contribute to systematic deviations from the experimental measurements.

Notably, the simulations demonstrate that plasticizers significantly reduce the *T*_g_ of PVC. Specially, PVC-DOP shows a *T*_g_ of 303.5 K, representing a 55 K reduction compared to that of pure PVC, which closely matches the experimental value of 308.5 K [14]. In contrast, PVC-SDH exhibits a *T*_g_ of 333.1 K, 11% higher than its experimental reference of 300 K [14]. While the simulated *T*_g_ values generally align with the experimental ranges, absolute differences may arise from variations in the experimental and simulation conditions. For instance, the *T*_g_ reduction rate for PVC-SDH in the simulations (10.01%) slightly exceeds the 7.62% reduction observed experimentally using a comparable proportion of SDH (28.5%) [19].

A further analysis of Figure 2 reveals that PVC blends with varying DOP/SDH ratios exhibit significant plasticizing effects. The *T*_g_ values are measured as 306.1 K, 316.1 K, 324.3 K, and 327.9 K for PVC-DOP/SDH (7.1:2.9), PVC-DOP/SDH (4.9:5.1), PVC-DOP/SDH (2.8:7.2), and PVC-DOP/SDH (1.4:8.6), respectively. These results indicate that the incorporation of DOP, SDH, or their mixtures effectively reduces the intermolecular interactions between PVC chains, thereby enhancing the polymer chain mobility and significantly lowering the *T*_g_ of the PVC system. This observation corroborates the findings from Li et al. [19], where both SDH and DOP demonstrated analogous plasticizing effects, and further supports the conclusions drawn in Yin et al.’s study [32].

### 3.2. The Mechanical Properties

Mechanical testing of the cells under “constant-strain” conditions was performed using the “Mechanical Properties” module. The elastic modulus (*E*) represents a material’s capacity to resist elastic deformation under uniaxial loading, while the shear modulus (*G*) reflects the ratio of shear stress to shear strain. Both parameters are essential for characterizing the mechanical properties of materials [33]. Assuming the polymer material is in a state of stress equilibrium, the *E* and *G* for isotropic materials can be determined using Lame’s constants (*λ*, *μ*), as shown in Equations (1) and (2).

(1) $$E=\frac{\mu (3\lambda +2\mu )}{\lambda +\mu }$$

(2) G=μ

(3) $$\lambda =\frac{1}{3}(C_{11}+C_{22}+C_{33})-\frac{2}{3}(C_{44}+C_{55}+C_{66})$$

(4) $$\mu =\frac{1}{3}(C_{44}+C_{55}+C_{66})$$

**Figure 3:**
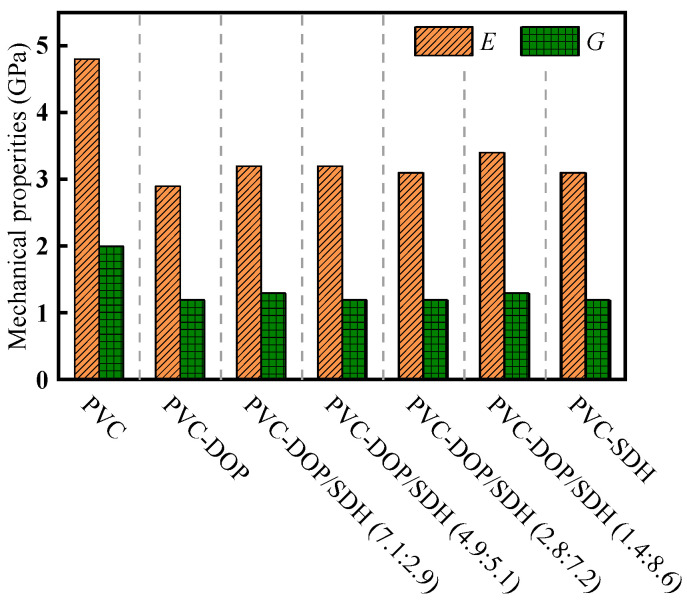
Effect of plasticizers on mechanical properties of PVC.

As shown in Figure 3, *E* and *G* of pure PVC are 4.85 GPa and 1.92 GPa, respectively. The addition of plasticizers leads to a substantial reduction in both parameters. Notably, the plasticizing effect of DOP is more pronounced compared to that of SDH. In the blended system of SDH and DOP, the incorporation of SDH did not lead to substantial alterations in the values of *E* and *G*.

### 3.3. The Solubility Parameter

The thermodynamic compatibility among polymer components is a critical factor in PVC processing. As shown in Table 3, the solubility parameter (*δ*) of PVC is 18.67 MPa^0.5^, which closely matches the experimental value, thereby validating the feasibility of the proposed methodology. The differences in the solubility parameters (Δ*δ*) between PVC, DOP, and SDH are all less than 2.0 MPa^0.5^, indicating excellent compatibility among these components [30]. Notably, SDH exhibits better compatibility with PVC than DOP.

### 3.4. PVC Chain Mobility

The *MSD* curves were analyzed to characterize the movement patterns of the PVC chains. The *MSD* values were calculated using Equation (5).

(5) $$MSD=\langle {\left|r_{i}\left(t\right)-r_{i}\left(0\right)\right|}^{2}\rangle$$

where *r*_i_(0) represents the initial position coordinates of atom *i*, while *r_i_*(*t*) denotes the position of atom *i* at time *t*. < > indicates the ensemble average over the entire system, calculated from the initial time *t* = 0.

It is well known that the slope of the MSD curve serves as a quantitative descriptor of the chain dynamics in PVC systems, where an increased slope steepness correlates strongly with enhanced molecular mobility.

**Figure 4:**
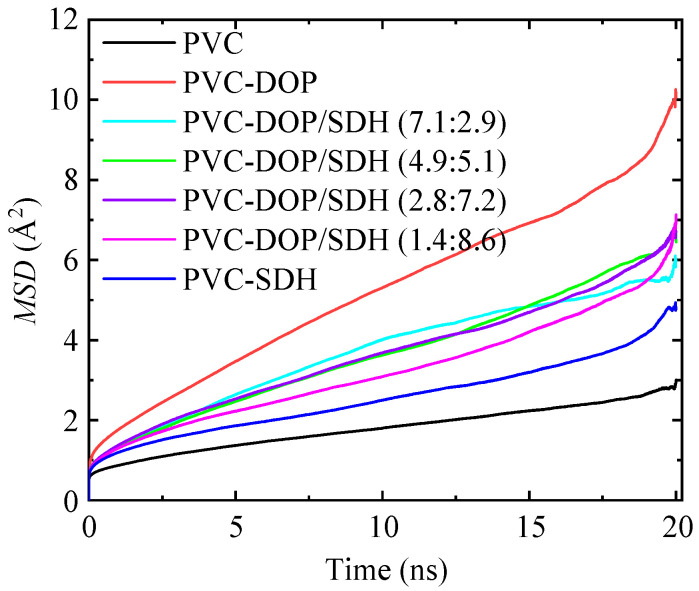
Comparison of *MSD* between PVC and plasticized PVC systems.

As shown in Figure 4, the MSD curve shows a good linear relationship with time in the middle section, but there are large fluctuations at the front and end of the curve. The reason for this lies in the imbalance at the front end of the curve and the accumulation of errors at the end of the curve. Pure PVC exhibits the smallest slope in its *MSD* curve among the composite systems, suggesting that the intermolecular interactions between PVC chains are the most pronounced. Consequently, these strong interactions significantly restrict the mobility of the PVC chains. In contrast, the plasticized PVC systems demonstrate steeper slopes in their MSD curves compared to those for pure PVC. This clearly demonstrates that the addition of plasticizers leads to a reduction in structural rigidity through molecular lubrication, an enhancement in the polymer chain flexibility, and the promotion of segmental dynamics, all of which collectively contribute to improving its processibility.

In pure PVC, strong van der Waals forces exist between the Cl atoms of the PVC chains, severely hindering the relative motion of the molecular chains. However, the insertion of DOP or SDH into PVC effectively mitigates the entanglement of the PVC chains, further increasing the distance between them and reducing the van der Waals forces. This leads to an enhancement in the plasticity of the PVC material. Moreover, the PVC chains in the PVC-DOP system move faster compared to those in the PVC-SDH system. Therefore, PVC chains are more mobile in the PVC/DOP composite, facilitating easier chain movement and contributing to improved plasticizing effects.

### 3.5. The Mechanism of the Mixed Plasticizers

*E*_Inter_ is an effective parameter for quantitatively characterizing the strength of the interactions between different substances. Binding energy is expressed as the following equation [38].

(6) $$E_{I\mathrm{nter}}=E_{\mathrm{AB}}-(E_{A}+E_{B})$$

where *E*_Inter_ represents the interaction energy between PVC chains, *E*_AB_ is the total energy of PVC, and *E*_A_ and *E*_B_ are the individual energies of the two PVC chains. *E*_Inter_ originates from non-bonding interactions (*E*_nonbond_). *E*_nonbond_ is mainly composed of van der Waals forces (*E*_vdW_) and electrostatic forces (*E*_elec_).

**Figure 5:**
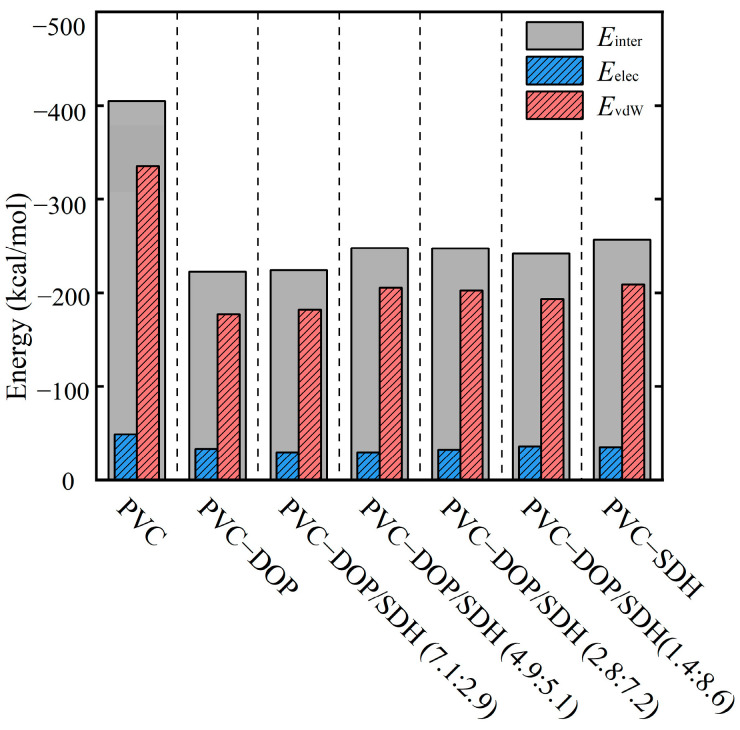
The binding energies of PVC chains at 298 K (kcal/mol).

To compare the interaction strength between PVC chains, cells were employed to calculate the binding energies. The calculated binding energies are summarized in Figure 5. As shown in Figure 5, the binding energy in the pure PVC system is measured to be the highest at 423.31 kcal/mol, whereas in the PVC-DOP and PVC-SDH systems, the binding energies are determined to be 233.11 kcal/mol and 255 kcal/mol, respectively. This indicates that the incorporation of plasticizers (DOP and SDH) weakens the intermolecular forces between the PVC chains, thereby reducing the compactness of the system. Specifically, the intermolecular interactions within the PVC chains are stronger in the pure PVC system compared to those in the PVC-DOP/SDH systems at the same mass ratio. The strength of the intermolecular interactions follows the order PVC > PVC-SDH > PVC-DOP/SDH (2.8:7.2) > PVC-DOP/SDH (4.9:5.1) > PVC-DOP/SDH (7.1:2.9) > PVC-DOP. A further analysis reveals that the interactions between polymer chains are predominantly governed by *E*_vdW_.

From a molecular conformation perspective, SDH contains more oxygen-containing functional groups compared to DOP, indicating a higher likelihood of hydrogen bond formation between SDH and PVC, which enhances their interaction. Furthermore, the stronger intermolecular interactions between PVC and SDH promote the formation of a physical cross-linking network structure in the PVC/SDH composite, resulting in improved mechanical properties. This is attributed to the presence of two oxygen atoms in the furan ring of SDH, which generate stronger attractions with the polar groups on the PVC molecules, resulting in stronger intermolecular forces. This also explains why the plasticizing effect of the PVC composites containing SDH is weaker than that in those containing DOP [19]. For the PVC system blended with both DOP and SDH, the interaction energy lies between those of the two individual components, and as the proportion of DOP increases, the interaction energy between the components decreases.

The differences in the intermolecular interactions between SDH or DOP and PVC also affect the free volume characteristics of the polymer system. The FFV, calculated to characterize the free volume of blended amorphous cells, can be determined using the following empirical equation [39].

(7) $$FFV=\frac{V_{F}}{V_{F}+V_{O}}\times 100\%$$

where *V*_O_ is the occupied volume of the polymer, and *V*_F_ is the free volume.

**Figure 6:**
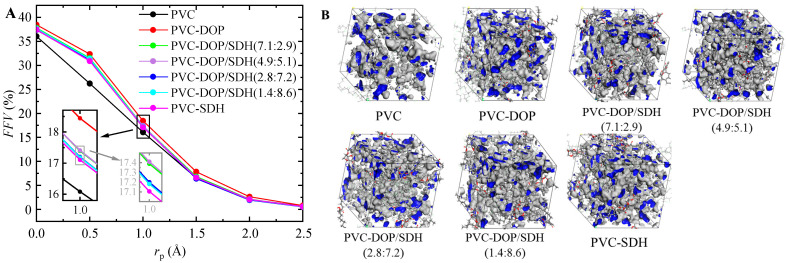
(**A**) *FFV*s of cells and (**B**) accessible free volumes (blue regions) of cells at *r*_p_ = 1.0 Å.

As shown in Figure 6, the simulation results indicate that the *FFV* of pure PVC cells is the lowest, whereas the *FFV* for PVC-DOP reaches its maximum value. A higher *FFV* facilitates the diffusion of plasticizer molecules within the system. Although the *FFV* values of the other groups are lower than that of PVC-DOP, these differences are not statistically significant. Importantly, the predictions derived from the *FFV* values are in agreement with prior conclusions and substantiate the research findings reported in reference [40]. Figure 6 presents the accessible free volumes for cells with a probe radius *r*_p_ = 1.0 Å, respectively.

**Figure 7:**
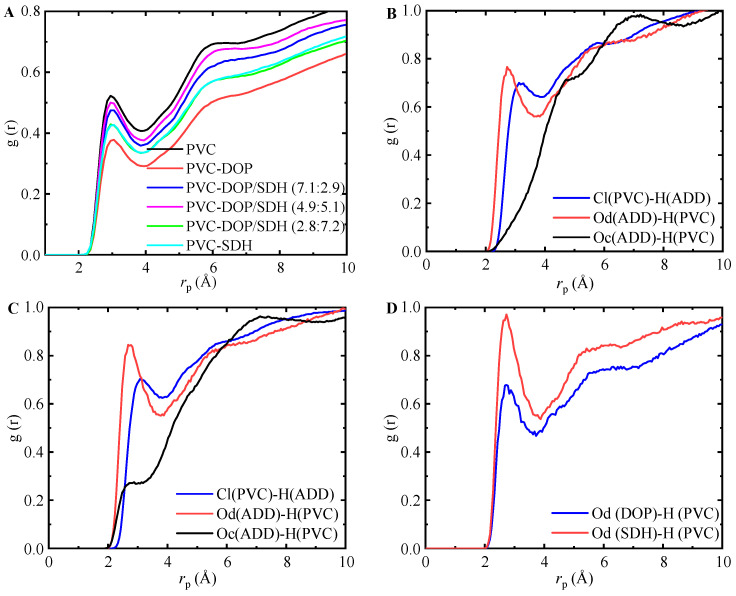
Site–site RDFs of PVC + PVC or plasticizer systems: (**A**) PVC; (**B**) PVC-DOP; (**C**) PVC-SDH; (**D**) PVC-DOP/SDH (4.9:5.1).

While the *FFV* analysis provides insight into the free volume characteristics and their impact on plasticization, a comprehensive understanding of the molecular interactions requires an analysis of the coordination between the plasticizer atoms and PVC sites. This approach can be implemented by examining site–site RDFs, as shown in Figure 7. Specifically, the chlorine atoms in PVC were designated as Cl (PVC), and hydrogen atoms were designated as H (PVC). In the plasticizers, the oxygen atoms in the carbonyl group (-C=O) were labeled as Od (ADD), Od (DOP), or Od (SDH), while the oxygen atoms in the ether group (C-O-C) were labeled as Oc (ADD).

The data demonstrate that these interactions are most prominent in the pure PVC cell, while they are substantially reduced in the PVC-DOP cell. This indicates that the incorporation of DOP significantly suppresses the formation of intermolecular hydrogen bonds among the PVC polymer chains. The hydrogen bonding interactions for the other cells are intermediate between those for pure PVC and the PVC-DOP cells, lacking discernible trends. The most probable intermolecular distances between PVC and DOP/SDH are approximately 2.85 Å and 3.05 Å, respectively. The initial peak at 2.85 Å indicates strong hydrogen bonding interactions between H (PVC) and Od (ADD). Additionally, the peak at 3.05 Å suggests the presence of a weak hydrogen bond between Cl (PVC) and H (ADD). Jagarlapudi et al. [23] proposed that the polar groups in plasticizers can enhance the plasticization of PVC by effectively disrupting the entanglement of PVC chains. Notably, all plasticizers demonstrate interactions between Od (ADD) and H (PVC). It is worth noting that the hydrogen bonding interactions between Od (DOP) and H (PVC) are the weakest. SDH exhibits significantly stronger hydrogen bonding interactions with PVC, suggesting a more robust interaction between SDH and PVC, which consequently hinders the diffusion of SDH.

### 3.6. Plasticizer Diffusion

Based on Equation (5), the *MSD* of the plasticizer’s molecular diffusion was analyzed further, and the diffusion coefficient of the plasticizer molecules was subsequently determined using Einstein’s Equation (8):

(8) $$D=\frac{1}{6N}\underset{t\to \infty }{\lim}\frac{d}{dt}\sum_{i=1}^{N}\langle {\left|r_{i}\left(t\right)-r_{i}\left(0\right)\right|}^{2}\rangle$$

where *D* refers to the molecular diffusion coefficient.

**Figure 8:**
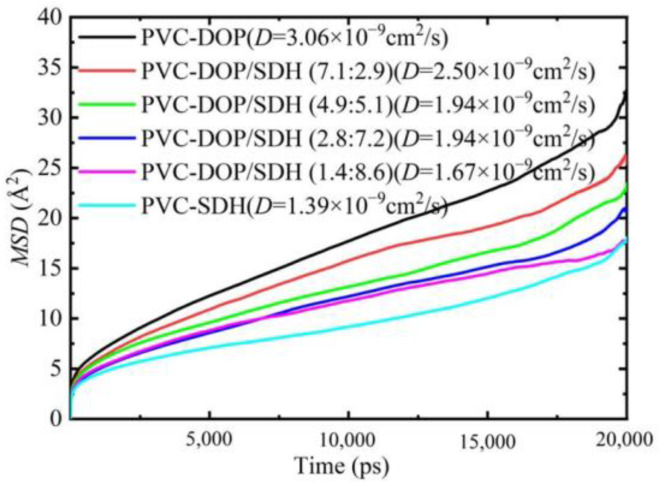
The *MSDs* and the diffusion coefficients of plasticizers in five cells.

We compared the diffusion coefficients of DOP, SDH, and the DOP/SDH mixture in the PVC/plasticizer system, as illustrated in Figure 8. The results indicate that the DOP plasticizer exhibits the highest diffusion capacity in the PVC-DOP cell, while SDH shows the lowest diffusion capacity in the PVC-SDH cell. In the PVC-DOP/SDH mixture cells, the average diffusion coefficient of the mixed DOP/SDH plasticizer also tends to decrease with an increasing SDH content. This reduction may be attributed to the stronger interactions between the bio-based plasticizer SDH and the PVC. SDH possesses more polar functional groups capable of forming hydrogen bonds with PVC compared to DOP, resulting in a lower diffusion capability than that of DOP. Overall, SDH demonstrates potential to be substituted for DOP in applications where migration resistance is critical. This result shows a good agreement with the analytical results on the free volume.

**Figure 9:**
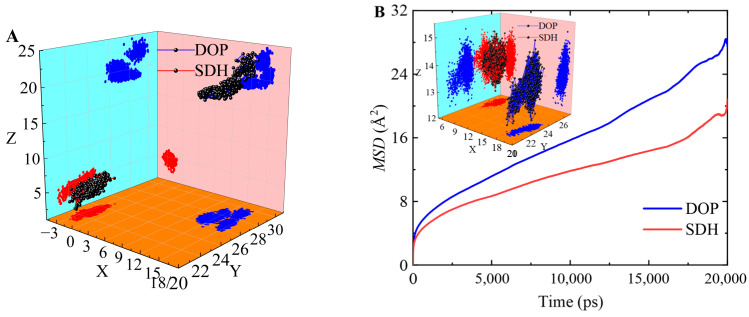
(**A**) The movement trajectories of DOP or SDH in the PVC-DOP/SDH (4.9:5.1) cell; (**B**) the *MSDs* and the movement trajectories of DOP or SDH in the PVC-DOP/SDH (4.9:5.1) cell.

The diffusion behavior of plasticizer molecules can also be observed from the trajectory of the plasticizer molecules in the PVC cells during the MD simulation. By analyzing the range of coordinates of the plasticizer molecules’ movement trajectories in these systems, it is easy to see that the distribution range for the plasticizer’s diffusion trajectory in the PVC-DOP cell is the widest and that the diffusion trajectories overlap densely in the PVC-SDH cell (Figure 9A). This phenomenon is in conformity with the results on the *MSD* and the *FFV* above, which further verifies the reliability of the results in this simulation. The movement trajectory can visually demonstrate the motion of the small molecules in polymers. From the trajectories of the plasticizer molecules in the PVC-DOP/SDH (4.9:5.1) cell, the diffusion behavior of the plasticizer molecules can also be observed (Figure 9B). It is easy to see that the diffusion trajectories of the SDH molecules densely overlap, while the DOP molecules show jumping phenomena.

## 4. Conclusions

This study was designed to optimize the formulations for partially replacing traditional plasticizers with bio-based alternatives. Through MD simulations, the feasibility and microscopic mechanisms of substituting DOP with SDH were systematically investigated, with a focus on compatibility, *T*_g_, mechanical properties, *E*_Inter_, *FFV*, and the RDF. The results indicate that PVC demonstrates excellent compatibility with both plasticizers, with SDH exhibiting a significantly superior compatibility compared to that of DOP. The incorporation of a DOP/SDH mixed plasticizer increases the mobility of the PVC chains, leading to a reduction in *T*_g_, *E*, and *G*, which consequently enhances the plasticizing effect. The migration resistance of SDH is superior to that of DOP. Partially replacing DOP with SDH can maintain the plasticizing effect while reducing the migration risk, thereby enhancing the safety of PVC materials. Future research should focus on the safety of mixed plasticizers in food-contact applications and refine the MD simulation cells further by increasing the number of PVC chains to improve the accuracy and reliability of the predictions.

## Figures and Tables

**Table 1:** Parameters and density of cells containing varying proportions of plasticizers.

Cells	Cell Component	DOP Mass Ratio (%)	SDH Mass Ratio (%)	Number of Atoms	Density (g/cm^3^)
PVC	2× PVC	0	0	1204	1.40
PVC-DOP	2× PVC + 14× DOP	30.43	0	2128	1.20
PVC-DOP/SDH (7.1:2.9)	2× PVC + 10× DOP + 4× SDH	21.69	8.85	2128	1.23
PVC-DOP/SDH (4.9:5.1)	2× PVC + 7× DOP + 7× SDH	15.17	15.48	2128	1.23
PVC-DOP/SDH (2.8:7.2)	2× PVC + 4× DOP + 10× SDH	8.65	22.08	2128	1.24
PVC-DOP/SDH (1.4:8.6)	2× PVC + 2× DOP + 12× SDH	4.32	26.47	2128	1.24
PVC-SDH	2× PVC + 14× SDH	0	31.37	2128	1.24

**Table 2:** Comparison of experimental and simulated glass transition temperatures for PVC systems.

	Simulation Value	Experimental Value	Our Work’s Value
*T_g_*	366.0 K [26], 368.6 K [30], 361.0 K [24]	356.05 K [8], 354.55 K [10], 356.15 K [12], 366.65 K [31]	359.3 K

**Table 3:** Prediction of miscibility of PVC with DOP and SDH through MD simulations.

Materials	*δ*_exp_	*δ*_sim_	*δ*_this work_
PVC	19.35 [34]	17.30 [35]	18.67
DOP	19.62 [36]	18.36 [37]	17.21
SDH	/	/	18.83

## Data Availability

The data are contained within the article.

## Supplementary Materials

The following supporting information can be downloaded at https://www.mdpi.com/article/10.3390/polym17121655/s1. Figure S1. Chemical structures of (A) DOP, (B) PVC, and (C) SDH. Figure S2. The total potential energy of the investigated systems during annealing simulation undergoing 10 cycles. Figure S3. Simulated cells with composition ratios of DOP and SDH. The gray line represents the PVC molecule chain, and the green ball represents DOP, while the pink stick represents SDH: (A) PVC; (B) PVC-DOP; (C) PVC-DOP/SDH (7.1:2.9); (D) PVC-DOP/SDH (4.9:5.1); (E) PVC-DOP/SDH (2.8:7.2); (F) PVC-DOP/SDH (1.4:8.6); and (G) PVC-SDH. Figure S4. Temperature and energy fluctuation curves for the cells during the NVT MD simulation (0–20 ns): (A) PVC; (B) PVC-DOP; (C) PVC-DOP/SDH (7.1:2.9); (D) PVC-DOP/SDH (4.9:5.1); (E) PVC-DOP/SDH (2.8:7.2); (F) PVC-DOP/SDH (1.4:8.6); and (G) PVC-SDH. Figure S5. Site–site RDFs of PVC + plasticizer systems: (A) PVC-DOP/SDH (7.1:2.9); (B) PVC-DOP/SDH (4.9:5.1); (C) PVC-DOP/SDH (2.8:7.2); and (D) PVC-DOP/SDH (1.4:8.6).

## Abbreviations

The following abbreviations are used in this manuscript:

*T*_g_	Glass transition temperature
*E*_Inter_	Interaction energy
*E*_elec_	Electrostatic interaction energy
*E*_vdW_	Van der Waals interaction energy
*E*	Elastic modulus
*G*	Shear modulus
*δ*	Solubility parameter
H	Hydrogen atom
C	Carbon atom
O	Oxygen atom
MD	Molecular dynamics
PVC	Polyvinyl chloride
DOP	Dioctyl phthalate
SDH	Isosorbide diheptanoate
PAEs	Phthalates
FFV	Fractional free volume
NVT	Canonical ensemble
NPT	Isobaric–isothermal ensemble
RDF	Radial distribution function
MSD	Mean squared displacement
PPPM	Particle–particle particle–mesh
Od (ADD)	The oxygen atoms in the carbonyl group (-C=O) of the additive
Od (DOP)	The oxygen atoms in the carbonyl group (-C=O) of dioctyl phthalate
Od (SDH)	The oxygen atoms in the carbonyl group (-C=O) of isosorbide diheptanoate
