# Supplementary material for: Molecular Dynamics Simulation of Plasticizing Effect of Mixed Dioctyl Phthalate and Isosorbide Diheptanoate on Polyvinyl Chloride Material
Source: Polymers (Basel). 2025 Jun 14;17(12):1655. doi: 10.3390/polym17121655 (PMC12197240; doi:10.3390/polym17121655)
Supplement: Supplementary file 1 [file polymers-17-01655-s001.zip › polymers-3684315-supplementary.pdf]

Figure S1. Chemical structures of (A) DOP, (B) PVC and (C) SDH.

Figure S2. The total potential energy of the investigated systems during Anneal simulation undergoing 10 cycles.

Figure S3. Simulated cells with composition ratios of DOP and SDH. (The gray-line represents PVC molecule chain, the green-ball represents DOP, while the pink-stick represents SDH: (**A**) PVC; (**B**) PVC-DOP; (**C**) PVC-DOP/SDH (7.1:2.9); (**D**) PVC-DOP/SDH (4.9:5.1); (**E**) PVC-DOP/SDH (2.8:7.2); (**F**) PVC-DOP/SDH (1.4:8.6); (**G**) PVC-SDH.

Figure S4. Temperature and energy fluctuation curves of cells during the NVT MD (0-20 ns): (A) PVC; (B) PVC-DOP; (C) PVC-DOP/SDH (7.1:2.9); (D) PVC-DOP/SDH (4.9:5.1); (E) PVC-DOP/SDH (2.8:7.2); (F) PVC-DOP/SDH (1.4:8.6); (G) PVC-SDH.

Figure S5. Site−site RDFs of PVC + plasticizer systems): (**A**) PVC-DOP/SDH (7.1:2.9); (**B**) PVC-DOP/SDH (4.9:5.1); (**C**) PVC-DOP/SDH (2.8:7.2); (**D**) PVC-DOP/SDH (1.4:8.6).

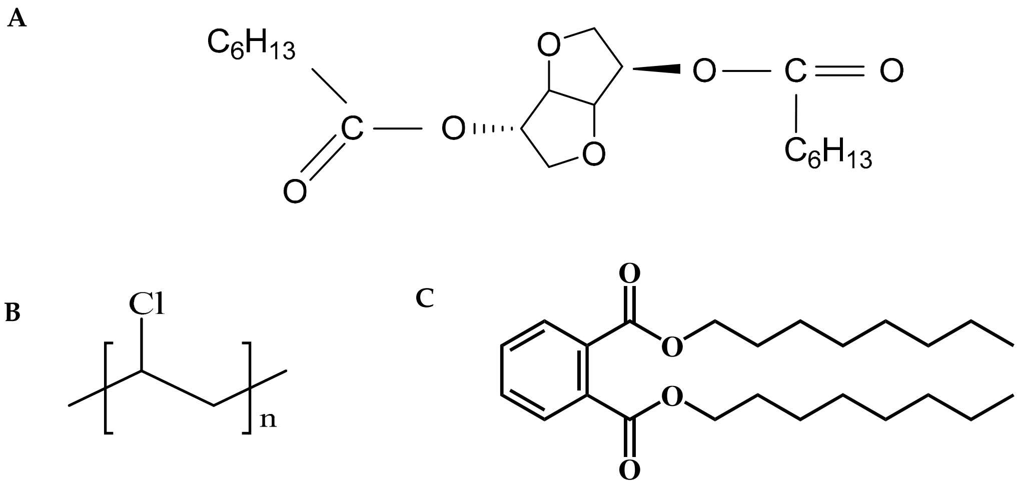

Figure S1. Chemical structures of (A) DOP, (B) PVC and (C) SDH.

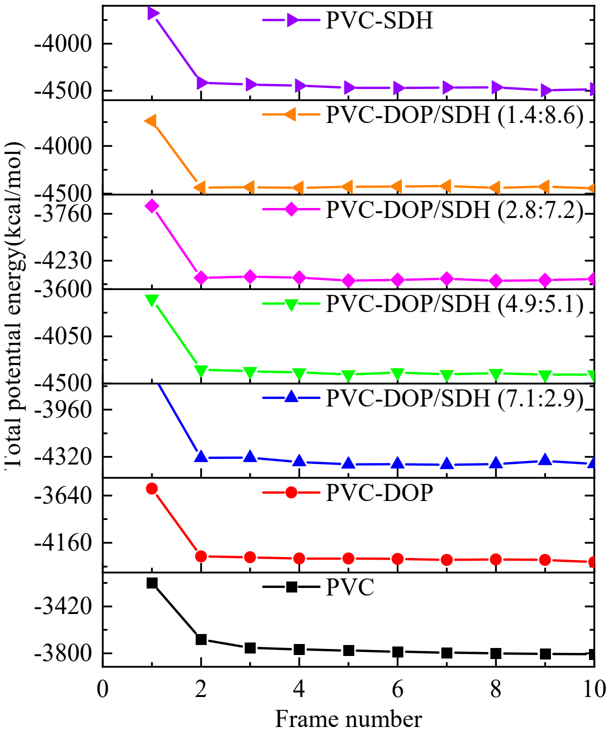

Figure S2. The total potential energy of the investigated systems during Anneal simulation undergoing 10 cycles.

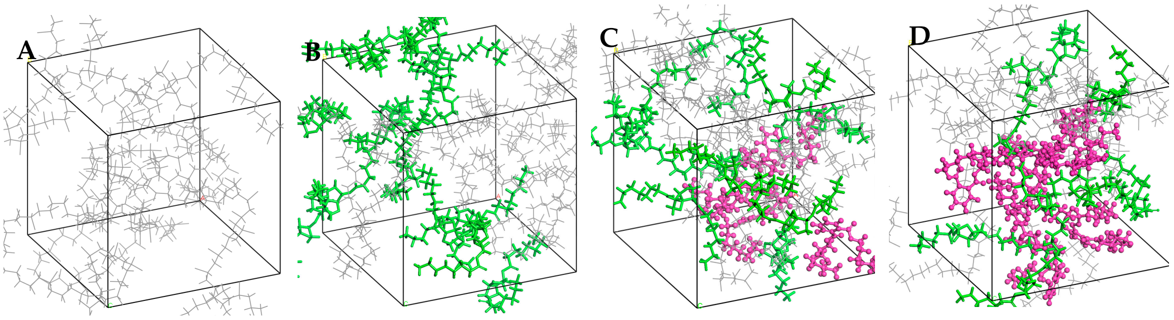

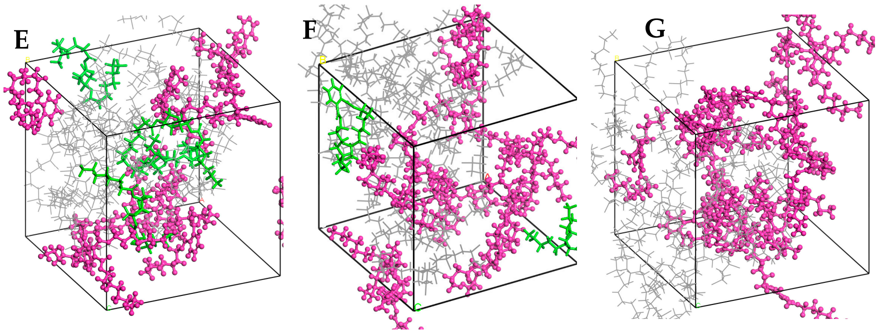

Figure S3. Simulated cells with composition ratios of DOP and SDH. (The gray-line represents PVC molecule chain, the green-ball represents DOP, while the pink-stick represents SDH: (**A**) PVC; (**B**) PVC-DOP; (**C**) PVC-DOP/SDH (7.1:2.9); (**D**) PVC-DOP/SDH (4.9:5.1); (**E**) PVC-DOP/SDH (2.8:7.2); (**F**) PVC-DOP/SDH (1.4:8.6); (**G**) PVC-SDH.

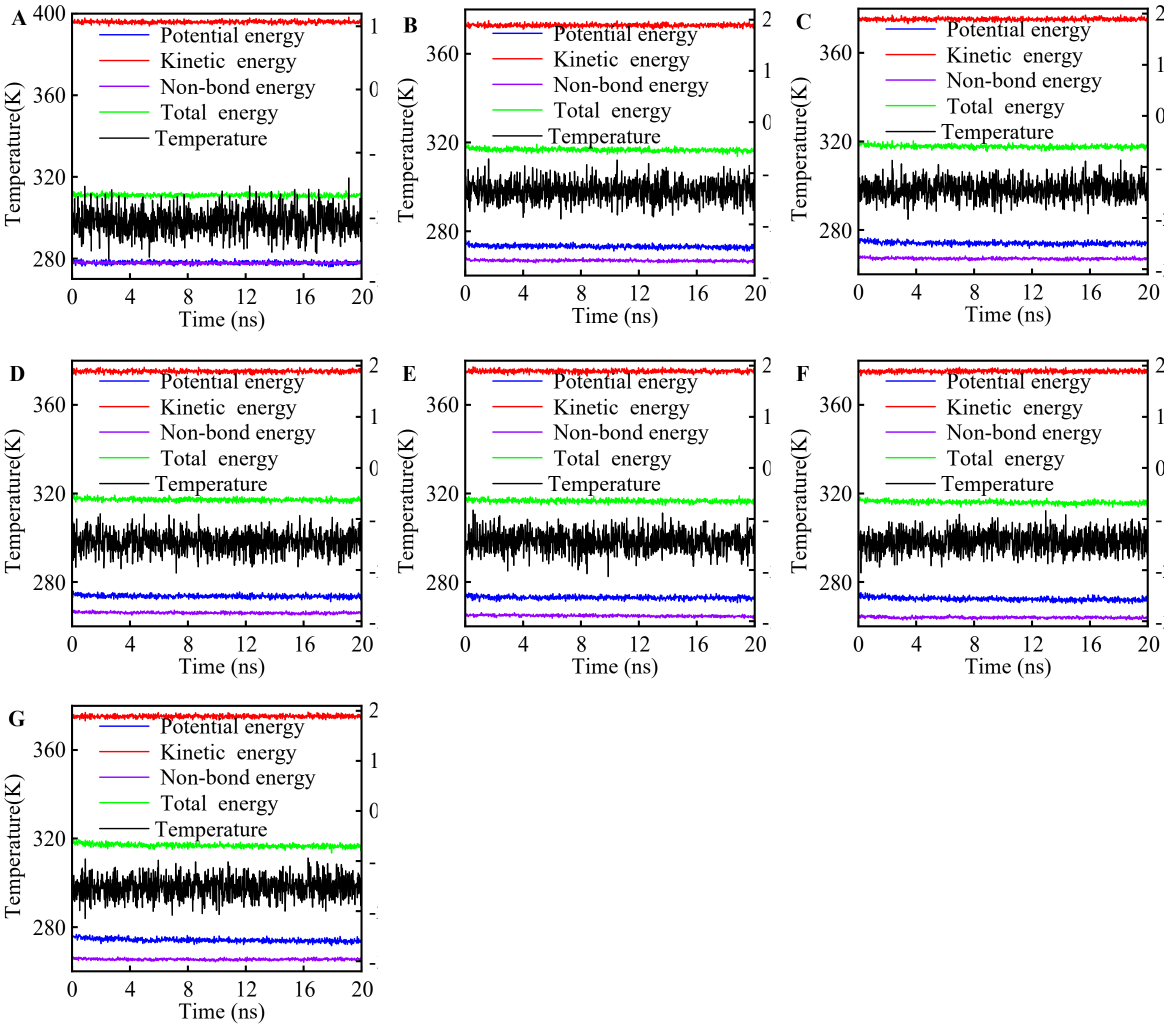

Figure S4. Temperature and energy fluctuation curves of cells during the NVT MD (0-20 ns): (**A**) PVC; (**B**) PVC-DOP; (**C**) PVC-DOP/SDH (7.1:2.9); (**D**) PVC-DOP/SDH (4.9:5.1); (**E**) PVC-DOP/SDH (2.8:7.2); (**F**) PVC-DOP/SDH (1.4:8.6); (**G**) PVC-SDH.

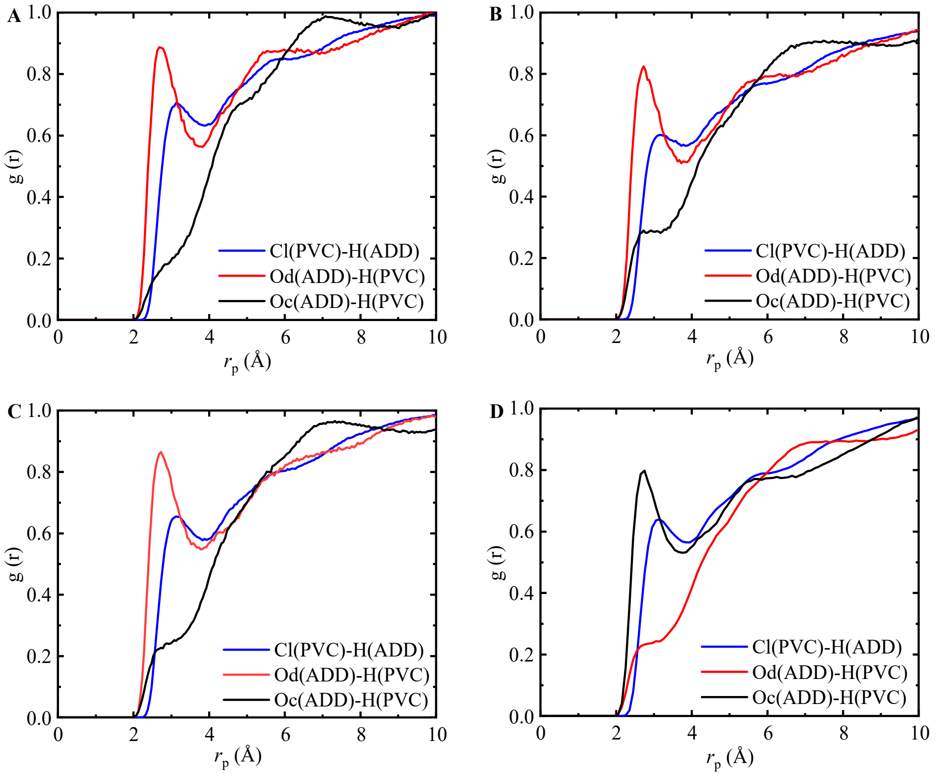

Figure S5. Site−site RDFs of PVC + plasticizer systems): (**A**) PVC-DOP/SDH (7.1:2.9); (**B**) PVC-DOP/SDH (4.9:5.1); (**C**) PVC-DOP/SDH (2.8:7.2); (**D**) PVC-DOP/SDH (1.4:8.6).
